# Management of bilateral adrenal myelolipoma without endocrine disorder: About a rare case report

**DOI:** 10.1016/j.eucr.2021.101755

**Published:** 2021-06-15

**Authors:** Ibrahim Boukhannous, Mehdi Chennoufi, Mohamed Mokhtari, Anouar El Moudane, Ali Barki

**Affiliations:** Department of Urology, Mohamed VI University Hospital Center, Mohamed I University, Oujda, Morocco

**Keywords:** Adrenal, Myelolipoma, Abdominal mass, Adrenalectomy

## Abstract

Adrenal gland myelolipomas are benign, hormonally inactive, and mostly asymptomatic and unilateral tumors. However, it could be symptomatic and bilateral in rare cases. The diagnosis is based on a CT scan and a histological study. We present a rare case of a surgically managed bilateral adrenal gland myelolipoma with a giant mass on the left side in a 40-year-old man who presented in our department for atypical abdominal pain. The patient underwent surgical resection of the left adrenal mass. Due to the resolution of the abdominal pain, a close follow-up for the right mass by CT scan was chosen.

## Introduction

Adrenal gland myelolipoma (AML) is a rare, benign, and nonfunctional tumor, firstly described in 1905.^1^ It is composed of mature adipous tissue associated with myeloid cells. 70% are small lesions and asymptomatic.^2^ In rare cases, it exceeds 10 cm and is then called giant, they can cause symptoms because of bulk effects or retroperitoneal hemorrhage due to spontaneous rupture. Only a few cases are bilateral. The present case report aims to describe a 40-year-old man with bilateral adrenal myelolipoma who initially present with atypical abdominal pain, and to discuss its management.

## Case report

A 40-year-old patient, without a particular medical history, was transferred to our urological unit for atypical abdominal pain. Clinical examination found a patient in good general condition, conscious, hemodynamically and respiratory stable, afebrile. Examination of the lumbar fossa found a slight tenderness of the left lumbar fossa. The ganglionic areas are free. The remainder of the physical examination is unremarkable.

**Fig. 1:**
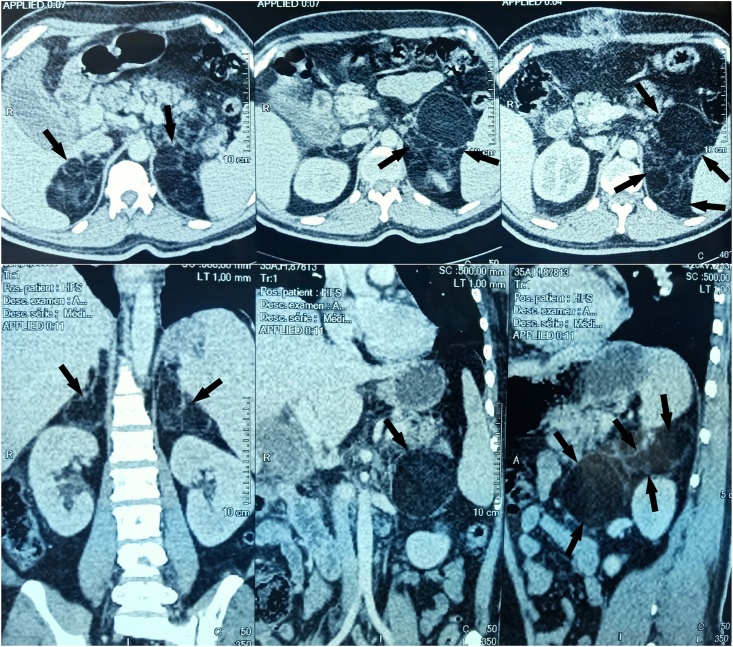
CT scan images showing bilateral adrenal masses of fatty density measuring on the right 74 mm and on the left 150 mm.

The computed tomography scan objectified bilateral adrenal masses of fatty density suggesting first myelolipoma measuring on the right 74 mm and on the left 150 mm (Fig. 1).

No abnormalities in biological assessment, as well as in hormonal investigation for adrenal masses; His serum level of ACTH, corticosteroid, and androgen were within the normal ranges; and his urine Metanephrine/normetanephrine was normal.

The patient underwent left adrenalectomy by subcostal incision. With a surgical time of 3 h without complications. The patient remained in the intensive care unit bed the first postoperative day. The patient was declared discharged after four days.

**Fig. 2:**
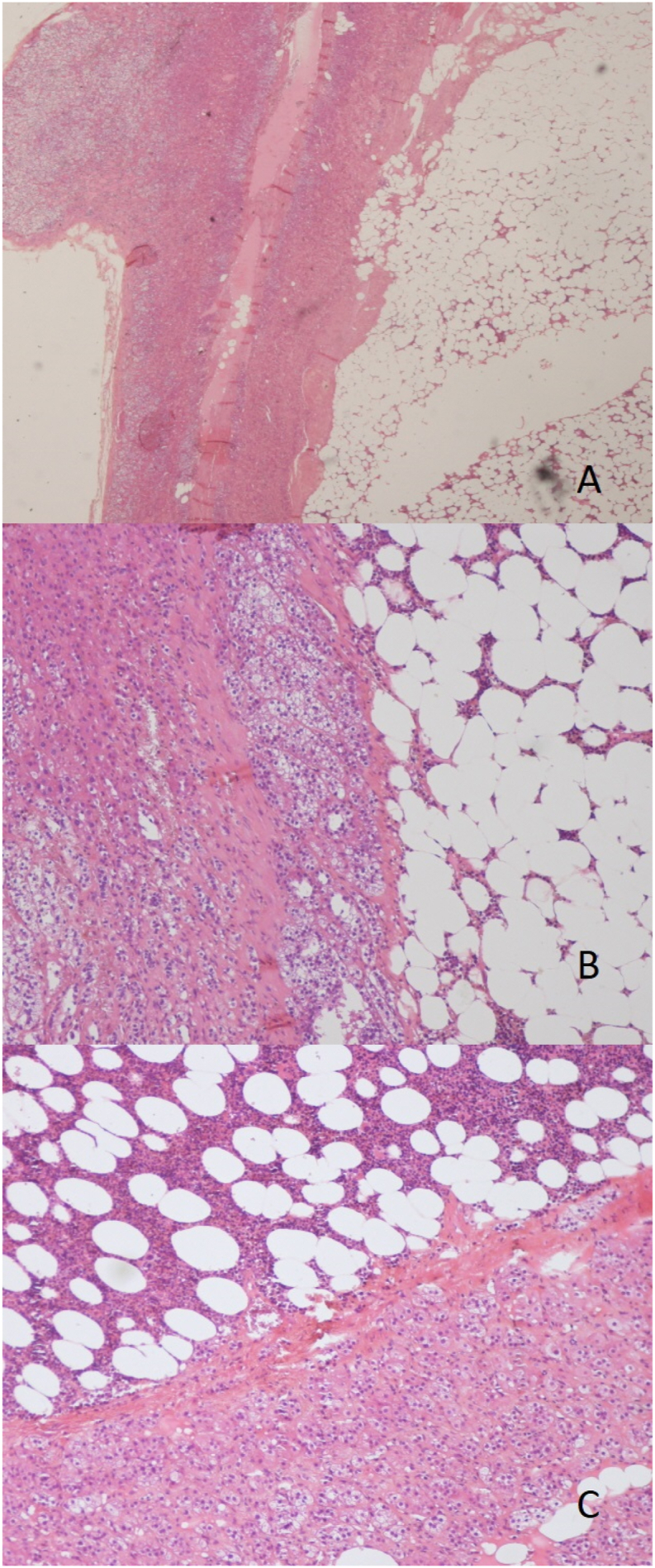
**A, B, C:** Microphotographs showing a neoplastic proliferation made of mature and variable-sized adipocytes with aggregates of hematopoietic elements, associated with adrenal gland tissue in the peripheral region.

The specimen measuring 15.1 cm and weighing 1432 g. Histopathological analysis with hematoxylin and eosin staining revealed a neoplastic proliferation made of mature and variable-sized adipocytes with aggregates of hematopoietic elements, associated with adrenal gland tissue in the peripheral region within the tumor confirming the diagnosis of adrenal gland myelolipoma (Fig. 2).

Due to the resolution of the abdominal pain, the clinical follow-up of the right adrenal myelolipoma was decided. On CT scan, there was no increase in the size of the right adrenal mass after 12 months, The patient remains in follow-up.

## Discussion

Adrenal myelolipomas are exceptional, benign, and non-functioning tumors composed of a mixture of mature adipose and hematopoietic cells. Their incidence increase with age, it represents 2% of all adrenal incidentalomas with three times more prevalent on the right side than on the left side because the right adrenal gland is easier to detect during abdominal ultrasonography, but improvements in medical imaging have increased the frequency of detection.^1^

Usually, adrenal myelolipomas are small, unilateral, and asymptomatic lesions incidentally detected when radiological investigations are done for other pathologies^1^^,^^2^ They are relatively slow-growing tumors. While big tumors can become symptomatic. Furthermore, giant myelolipoma can develop in rare cases with a size exceeding 10 cm in diameter. Symptoms can combine nonspecific abdominal pain, vomiting, constipation, hematuria, or renovascular hypertension due to compression of peritumoral tissue or intratumoral hemorrhage.^1^^,^^2^

The etiology of adrenal myelolipoma remains imprecise. There are several hypotheses including and degeneration of epithelial tissues of the adrenal cortex, extramedullary hematopoiesis due to the autonomous proliferation of bone marrow cells transferred during embryogenesis, and the most widely accepted theory is the adrenocortical cell metaplasia of the reticuloendothelial cells due to chronic stimulation such as inflammation, infection, necrosis, or stress.^2^^,^^3^

The ultrasound of an adrenal myelolipoma typically shows as a well-defined tumor with hyperechoic regions corresponding to adipose tissue and hypoechoic parts of the myeloid component. In 90% of the cases, the diagnosis of AML can be established by using a CT scan which is reported as the most sensitive diagnostic test for AML. It shows a radiolucent, avascular, and well-circumscribed mass that contains variable amounts of soft tissue and macroscopic fat with a negative Hounsfield Unit. Yet, in MRI, the AML appears as high-signal regions in T1 and T2. In contrast with the myeloid component which appears as low-signal in T1 and moderate signal in T2.^1^^,^^3^^,^^4^

There are no specific guidelines for the management of AML, and the decision should be taken in a multidisciplinary setting on a case-by-case basis. In general, bilateral adrenalectomy should be avoided to conserve hormonal function. Furthermore, Small tumors should be managed by monitoring over 1–2 years with imaging controls. On the other hand, in case of symptoms, significant growth, or hormonal activity, surgical removal is indicated by laparotomy or laparoscopy. Also, large masses of a size bigger than 8 cm should be removed, given their great potential of spontaneous rupture causing a retroperitoneal hemorrhage.^1^^,^^3^^,^^5^

## Conclusion

We reported an extremely rare case of a bilateral AML with a giant neoplasm in the left side accompanied by abdominal pain in a patient without an endocrine disorder. However, the endocrine function must be preserved when possible. The surgical treatment of the asymptomatic lesions is not obligatory even if they exceed 7 cm, because of the very low risk of rupture, and a close follow-up strategy could be used. The management should be discussed on a case-by-case basis. More case reports and long-term studies are needed to establish clear guidelines for the management of bilateral AML.

## Declaration of competing interest

None of the contributing authors have any conflict of interest, including specific financial interests or relationships and affiliations relevant to the subject matter or materials discussed in the manuscript.
